# The *Biomolecules* Journal Club: Highlights on Recent Papers—1

**DOI:** 10.3390/biom12010086

**Published:** 2022-01-06

**Authors:** Francesc Rabanal, Mark S. Johnson, Alessandro Alaimo, Victor M. Bolanos-Garcia, Travis Beddoe

**Affiliations:** 1 Organic Chemistry Section, Department of Inorganic and Organic Chemistry, Faculty of Chemistry, University of Barcelona, 08028 Barcelona, Spain; 2 Structural Bioinformatics Laboratory, Biochemistry, Faculty of Science and Engineering, Åbo Akademi University, Tykistökatu 6A, 20520 Turku, Finland; 3 Department of Cellular, Computational and Integrative Biology (CIBIO), University of Trento, Via Sommarive 9, 38123 Trento, Italy; 4 Department of Biological and Medical Sciences, Faculty of Health and Life Sciences, Oxford Brookes University, Oxford OX3 OBP, UK; 5 Department of Animal, Plant and Soil Sciences and Centre for AgriBioscience, La Trobe University, Bundoora, VIC 3083, Australia

## 1. Introduction

We are glad to share with you our first Journal Club and to highlight some of the most interesting papers published recently. We hope we will tease your curiosity and encourage you to read full papers outside of your research area, which you may not have read otherwise.

The *Biomolecules* Scientific Board wishes you an exciting read.

## 2. Interaction Mode of the Novel Monobactam AIC499 Targeting the Penicillin Binding Protein 3 of Gram-Negative Bacteria

### Highlight by Francesc Rabanal

The need of continuous research and development in the field of antibiotics to fight antimicrobial resistance is a well-known fact. In a recent *Biomolecules* article, Freischem, Weiergräber and co-workers reported the X-ray structures of covalent intermediates (long-lived acyl-enzyme intermediates) formed between a novel β-lactam antibiotic (AIC499) and the penicillin binding protein 3 (PBP3) from *Escherichia coli* (Ec) and *Pseudomonas aeruginosa* (Pa) [1]. The apo structures of Pa-PBP3 and of a newly designed TPd construct featuring Ec-PBP3 were also determined. In addition, NMR studies were performed using 2D ^1^H-^15^N TROSY-HSQC spectroscopy on a spectrometer with modest field strength (700 MHz).

The structural data presented helps to rationalize the binding mechanism of this antibiotic and provides new insights into various aspects of PBP3 structure and dynamics. Altogether, these results could ultimately be of use to further improve the properties of AIC499.

Background: AIC499 is a monobactam developed by Aicuris that in combination with a β-lactamase inhibitor displays broad and potent antibacterial activity against Gram-negative pathogens, including multi-drug resistant strains, and improved resistance to most classes of β-lactamases. AIC499 is in clinical development, and a the first-in-human phase 1 trial is currently ongoing and being supported by the European Innovative Medicines Initiative (IMI) within the COMBACTE-MAGNET project.

## 3. First Crystal Structure of PGM5, an Inactive Member of the Phosphoglucomutase Family

### Highlight by Mark S. Johnson

The phosphoglucomutase (PGM) enzyme family fulfils an essential role in energy metabolism by reversibly exchanging a phosphate group between the 1 and 6 position on glucose, thus providing the required intermediates linking the essential metabolic pathways of glycolysis and gluconeogenesis with glycogen synthesis and glucose release from glycogen stores. Not surprisingly, PGMs are found where glycogen is stored and energy metabolism is a central function, i.e., in the liver and muscle.

In their article, Gustafsson et al. [2] report the first three-dimensional structures of PGM5 obtained using X-ray diffraction, and for the Atlantic and Baltic Sea herring. PGM5 in herring is associated with ecological acclimatization to the Baltic waters after the last glaciation event some 10,000 years ago.

PGM5 was earlier identified as a muscle protein without enzymatic activity and the research reported in the current article is intriguing from several points of view. PGM5 not only retains a high similarity to the enzymatically active PGM1 enzyme in humans, but is enzymatically inactive (although, interestingly, crystals soaked with glucose-1,6-bisphosphate resulted in bound glucose-1-phosphate); instead, PGM5 has an alternative non-enzymatic function, acting to structurally bind other proteins as part of the muscle.

Gustafsson et al. [2] compare the structures within the PGM family and propose the likely reasons for the lack of catalytic activity of PGM5, as well as proposing a role for the single alanine to valine mutation that largely distinguishes the high salinity Atlantic herring and low salinity Baltic Sea herring.

## 4. Glycosphingolipids: A New Hope in Prostate Cancer Diagnosis

### Highlight by Alessandro Alaimo

Since its introduction, about 30 years ago, the prostate specific antigen (PSA) blood test has been the most widely used, non-invasive method for prostate cancer (PCa) diagnosis. Nevertheless, over the years, this test has proven to be poorly accurate since increased plasmatic levels of PSA can be associated to cancer unrelated factors, such as age, local inflammation, or urinary infections. This lack of specificity has often created difficulties in distinguishing indolent from aggressive PCa, with consequent over-diagnosis and the overtreatment of patients. Therefore, the search for more reliable biomarkers to detect and recognize low- versus high-risk PCa cases remains a priority. The advent of *Omics* technologies has led to the identification of potential new biomarkers that can complement the role of PSA for PCa managing. Among them, circulating plasma lipids are emerging as important players in PCa diagnosis.

In an interesting paper published in *Biomolecules*, Snider and colleagues used a metabolomic approach to analyze plasma samples obtained from both African-American and European-American patients, to identify metabolites related to aggressive PCa [3]. The authors reported that fifteen plasma lipids associated with PCa aggressiveness were sphingolipids. Among them, glycosphingolipids, which included tetrahexosylceramide and trihexosylceramide, proved to be strongly associated with cancer aggressiveness. Further metabolomic analysis, aimed at finding race/ethnicity-specific metabolites associated with PCa status, revealed two compounds exclusively associated with European-American men, whilst twenty-four metabolites were associated with PCa severity only in African-American patients. Finally, the TGCA database was consulted to explore whether there were aberrations in the genes implicated in glycosphingolipid metabolism. The data obtained, highlighted alterations not only in glycosphingolipid genes in PCa, but also in lung squamous carcinoma, ovarian and other cancers.

Overall, this study unveils that specific plasma glycosphingolipids are strongly associated with PCa, suggesting their potential use in diagnostic to establish PCa aggressiveness across the racial population.

## 5. Structural Basis of Specific Glucoimidazole and 2 Mannoimidazole Binding by the Enzyme β-Glucosidase Os3BGlu7 from Rice

### Highlight by Victor M Bolanos-Garcia

β-glucosidases and β-mannosidases hydrolyze substrates that differ slightly in structure (namely, they vary only in the stereochemistry at the C-2 position). Despite this knowledge, important details of substrate stereo selectivity by β-glucosidases and β-mannosidases remain to be fully understood. Nutho and collaborators addressed this important problem and reported the structural data of the enzyme β-glucosidase Os3BGlu7 from rice (Oryza sativa), complemented with molecular modeling investigations of Os3BGlu7 β-glucosidase interaction with the epimeric inhibitors glucoimidazole (GIm) and mannoimidazole (MIm) [4].

The crystal structure Os3BGlu7 β-glucosidase in a complex with GIm and solved at 2.30 Å, revealed that the epimeric inhibitor GIm was tightly bound in the -1 subsite of Os3BGlu7 β-glucosidase, while MIm was not. The 3D enzyme structure also showed that the intermolecular hydrogen bonding of the inhibitor to the enzyme active site plays a key role in complex formation. Although it was not possible to solve the 3D structure of Os3BGlu7 β-glucosidase in a complex with MIm, the use of closely related protein structures enabled the authors to carry out docking and molecular modeling simulations, which unveiled distinct conformational transitions for the epimeric inhibitors. Taken together, the studies suggest that a balance between distortion and binding energy is an important determinant of substrate specificity, and that different transition states are implicated in the hydrolysis of glucosides and mannosides by this class of glycoside hydrolases.

## 6. Pathogen Recognition of Sialic Acids

### Highlight by Travis Beddoe

Sialic acids (Sias) are sugar moieties that are present at the terminal end of glycans, which are important determinants in mediating a range of important biological interactions. The two common forms of Sias, Neu5Ac and Neu5Gc, which aid pathogen binding, are species-specific with humans not having the ability to synthesis Neu5Gc as the result of losing CMP-Neu5Ac hydrolase. The loss of CMP-Neu5Ac hydrolase has evolved due to certain pathogens. In a recent issue of *Biomolecules*, Patrycja Burzynska and co-workers reviewed the role of sialic acids as receptors for pathogens [5].

The authors reviewed all the pathogens that interact with Neu5Ac and Neu5Gc, which included viruses, bacteria and protozoa, highlighting that many of the pathogens can bind both Neu5Ac and Neu5Gc; however, several pathogens have adapted to bind one form of sialic acid. In particular, *Plasmodium* spp. have a range of sialic acid-binding proteins, such as EBA-175, EBA-140 and EBA-165, which all bind Neu5Gc to allow the invasion of red blood cells. However, *P. falciparum*, which infects humans, have gained the ability to bind Neu5Ac as well in response to the loss of Neu5Gc in humans. This arms race between host and pathogens has resulted in pathogens that require Neu5Ac, for infection to expand in humans. This highlights that a better understanding of Sias involvement in pathogenesis can allow for the development of novel therapies.
